# The Ethiopian Field Epidemiology and Laboratory Training Program: strengthening public health systems and building human resource capacity

**Published:** 2011-12-14

**Authors:** Daddi Jima, Getnet Mitike, Zegeye Hailemariam, Alemayehu Bekele, Adamu Addissie, Richard Luce, Peter Wasswa, Olivia Namusisi, Sheba Nakacubo Gitta, Monica Musenero, David Mukanga

**Affiliations:** 1 Ethiopian Field Epidemiology and Laboratory Training Program, P.O.Box 12953, Addis Ababa, Ethiopia; 2 African Field Epidemiology Network, P. O. Box 12874, Kampala, Uganda

**Keywords:** Ethiopia, Public health training, field epidemiology training program

## Abstract

The Ethiopian Field Epidemiology and Laboratory Training Program (EFELTP) is a comprehensive two-year competency-based training and service program designed to build sustainable public health expertise and capacity. Established in 2009, the program is a partnership between the Ethiopian Federal Ministry of Health, the Ethiopian Health and Nutrition Research Institute, Addis Ababa University School of Public Health, the Ethiopian Public Health Association and the US Centers of Disease Control and Prevention. Residents of the program spend about 25% of their time undergoing didactic training and the 75% in the field working at program field bases established with the MOH and Regional Health Bureaus investigating disease outbreaks, improving disease surveillance, responding to public health emergencies, using health data to make recommendations and undertaking other field Epidemiology related activities on setting health policy. Residents from the first 2 cohorts of the program have conducted more than 42 outbreaks investigations, 27analyses of surveillance data, evaluations of 11 surveillance systems, had28oral and poster presentation abstracts accepted at 10 scientific conferences and submitted 8 manuscripts of which 2are already published. The EFELTP has provided valuable opportunities to improve epidemiology and laboratory capacity building in Ethiopia. While the program is relatively young, positive and significant impacts are assisting the country better detect and respond to epidemics and address diseases of major public health significance.

## Introduction

Field Epidemiology and Laboratory Training Program (FELTPs) are competency-based training programs that help build capacity of countries to support and strengthen public health systems and infrastructure [1]. These programs support trainees, also called FELTP residents to develop their skills through hands-on application of epidemiology to real public health issues. For the most part, residents carry out research projects in priority areas of the districts they are attached, often under direct supervision of the local or provincial health leaders [2]. In Africa, these programs formed a networking alliance called the African Field Epidemiology Network (AFENET) in 2005, which consists of 12 programs spread across 20 countries, including Ethiopia [3].

The Ethiopian Field Epidemiology and Laboratory Training Program (EFELTP) is a comprehensive two-year competency-based post-graduate training and service program designed to build sustainable public health expertise and capacity. Established in February, 2009, EFELTP is a partnership between the Ethiopian Federal Ministry of Health (FMOH), the Ethiopian Health and Nutrition Research Institute, Addis Ababa University School of Public Health, the Ethiopian Public Health Association (EPHA), the US Centers of Disease Control and Prevention [4,5].

The vision of the program is to create a world class public health system that can support public health surveillance and response to health emergencies in Ethiopia. Its mission is to produce highly trained, competent health professionals that will strengthen public health surveillance and emergency response and improve health in the country [6].

### Country context

While progress has been made in recent years to improve health indicators, there is a need for improved capacity among public health professionals, particularly in the area of field epidemiology and creating a public health culture of using data for health decision making. Ethiopia has poor health indicators that necessitate for concerted efforts from all health service providers including epidemiologists and other public health providers. Expanded Programme for Immunisation (EPI) coverage, as calculated based on DPT3, stands at 73% [7] and antenatal coverage stands at just over half (52.6%). Ethiopia has one of the lowest life expectancy rates of 41 and 42 years for males and females respectively [8]. The country also has a high maternal mortality ratio of 673/100,000 live births while the infant mortality rate stands at 77/1000 [9]. Ethiopia is at constant risk of disease outbreaks such as measles due to low vaccination coverage, diarrheal and water borne diseases due to poor sanitation and water coverage and malnutrition mostly due to recurrent famines and droughts. Ethiopia also receives an influx of refugees from war torn Somalia and routinely faces both made-made and natural disasters like conflicts and droughts. The country also ranks 7^th^ highest among countries with the highest TB burden globally [10].

### Roles of partners in program

Each of the program partners has specific roles they play in the program. The FMOH and Regional Health Bureaus provide training field sites, field supervisors and deploy residents in support of their investigations and activities. Addis Ababa University School of Public Health is responsible for academic content of the program, designing a curriculum, providing classroom and didactic courses, and granting the program's degree. The EPHA channels funding, provides administrative support, and manages materials, supplies, logistics, and travel. The US CDC provides technical assistance and funding through the President′s Emergency Plan for AIDS Relief (PEPFAR).

In 2009, EFELTP joined the AFENET Network. By being in the latter, the program benefits from both technical and financial support, and closer linkages with other programs in the Network and all of AFENET's partners. The program has a country liaison officer based at the AFENET Secretariat who coordinates EFELTP activities in the network.

### Description of program

The EFELTP program offices are based in Addis Ababa, Ethiopia. The School of Public Health is the hosting institution for the program which is housed in at the Tikur Anbessa Hospital. However, a larger facility with more rooms for teaching, a computer laboratory, library and office is located on the premises of the Zewditu Memorial Hospital.

Residents spend about 25% of their time undergoing didactic training and the remaining 75% in the field working within the Federal Ministry of Health and Regional Health Bureaus where they investigate disease outbreaks, improve disease surveillance, respond to public health emergencies, and use health data to make recommendations on setting health policy for the nation [3].

There are five field sites (field bases) which are: Bahirdar Field Base (Amhara National Regional State), Mekele Field Base (Tigray Regional State), Oromyia Field Base (Oromyia Regional State), Hawassa Field Base (Southern Nations and Nationalities People's Region), and Addis Ababa PHEM (Public Health Emergency Management) Field Base located at the Ethiopian Health and Nutrition Research Institute in the Federal Ministry of Health.

### Course offered by the program

The program offers courses in epidemiology, biostatistics, surveillance, communications and scientific writing, computer application in public health, public health laboratory methods and bio-safety, health leadership and management and disaster management (Table 1). During residency, residents conduct a number of activities such as outbreak investigations, analysis of surveillance data, evaluation of surveillance systems, participate in collaborative research projects with numerous partners, develop abstracts, manuscripts, and oral or poster presentations for scientific conferences. The findings of such activities are useful in guiding policy and decision making within the FMOH and the respective regional health bureaus. After the training, the residents are awarded a Master of Public Health in Field Epidemiology by the Addis Ababa University School of Public Health.

**Table 1:** Course distribution for The Ethiopian Field Epidemiology and Laboratory Training Program (EFELTP)

N	Subject	ECTS (European Credit Transfer System)
1	Basic Epidemiology and Biostatics	3.5
2	Field Epidemiology	1.75
3	Public Health Surveillance	1.75
4	Public Health Lab methods and bio-safety	1.75
5	Communication and scientific writing	1.75
6	Computer application in Public Health	1.75
7	Management and leadership	1.75
8	Advanced Epidemiology and Epidemiology of priority health problems in Ethiopia	3.5
9	Disaster management	1.75
10	Field Residency I and II	26.25
11	Projects I and II	37
	**Total**	**24**

## Achievements and highlights of the program

### Enrolment and graduation

The program has enrolled three resident cohorts as of October 2011. Thirteen residents were enrolled in the first cohort in 2009, 22 in the second and 18 in the third cohort of 2011. The first cohort graduates received their degrees in July 2011. They returned to their sponsoring regions where they were re-assigned to new positions where they will use their newly acquired skills to support important public health response and surveillance activities. Such positions include becoming a regional head of Public Health Emergency Management units and regional heads of Integrated Disease Surveillance and Response (IDSR) responsible for disease investigation and surveillance.

### Outbreak investigations done

EFELTP residents have been involved in a number of outbreak investigations during their training. Residents have conducted outbreak investigations on multiple diseases including diarrheal disease, measles, meningitis, whooping cough, rabies, anthrax, vaccine-derived poliovirus, severe malnutrition, drinking water quality, motor vehicle accident surveillance, and nutritional surveillance. During these outbreaks, residents characterized the outbreaks, investigated the sources of the epidemics, offered health education and community sensitization and participated in immunisation campaigns.

### Surveillance activities done

Residents have also been involved in revising disease case definitions, disease reporting guidelines and forms. They have participated in Influenza A quarantine efforts at the international airport, and have routinely furnished the Ministry of Health with disease surveillance updates during outbreaks. Many of their recommendations from their investigations have been implemented including the adoption of mandatory safety belt laws, and provision of water and sanitary facilities during large gatherings at religious and cultural events.

### Conferences attended and publications

Most of the first cohorts have participated in the annual national EPHA Conference, the Ethiopian Medical Association annual conference, the AFENET Regional Conference in Mombasa, Kenya in 2009 and TEPHINET conference in Cape Town, South Africa in 2010 where residents made oral and poster presentations. The program has also started publishing research papers. A summary of the key achievements of the program is shown in Table 2. Among the new developments at the program is the introduction of a training course on Global Information Systems (GIS). The program also plans to expand to include at least two more field sites and possibly two other universities.

**Table 2:** Summary of Program outputs (Cohort 1 residents and first 12 months of cohort 2 residents)

	Achievements	Number
1	Outbreaks investigations and response	42
2	Surveillance data analysis	27
3	Surveillance systems evaluated	11
4	Collaborative and other public health projects	23
5	Accepted abstracts at conferences	28
6	Program submissions for publication	8
7	Manuscripts already published	2

### Challenges faced by program

The program has faced a number of challenges. Such constraints include providing adequate mentoring and supervision to residents at regional field bases, challenges with communication and access to information, and lack of transportation to support residents in field work and investigations.

## Conclusion

The EFELTP has provided noticeable improvement in the quality of public health practice in the country. The use of data and improved ability to use the tools of epidemiology to find evidence based answers to urgent health problems is most notable. While the program may be in its early stages, it has scored some positive and significant impacts which are assisting the country to better detect and respond to epidemics and address major public health challenges. Continued engagement and involvement of all stakeholders and program partners is necessary to ensure sustainability and expansion of the program beyond its current scope. Better coordination with public health related laboratory systems will further enhance the program's impact and additional resources to support field placement and supervision are necessary. The ministry of health should also ensure that priority is given to the program graduates and alumni if Ethiopia is to maximally benefit from the competencies that the program can offer.
